# Time-Domain Analysis of Chest Impedance for Venous Air Embolism Detection: An Open-Source Software Tool and Single-Cadaver Pilot Study

**DOI:** 10.7759/cureus.92317

**Published:** 2025-09-14

**Authors:** Chris Marcellino, Matt Johnston, Nathaniel Robinson, Arnoley S Abcejo

**Affiliations:** 1 Department of Anesthesiology and Perioperative Medicine, Mayo Clinic, Rochester, USA; 2 Department of Anesthesiology, University of Minnesota, Minneapolis, USA; 3 Department of Vascular Surgery, Mayo Clinic, Eau Claire, USA

**Keywords:** air embolism, chest impedance, intraoperative monitoring, open-source, python, vae

## Abstract

Venous air embolism remains one of the leading intraoperative neurosurgical complications and causes of intraoperative neurosurgical death. While transesophageal echocardiogram and precordial Doppler are commonly used in neurosurgical cases to detect entrained air, both have significant limitations, and neither tool has been adapted to automated notification. We theorize that (1) higher frequency variations (relative to the respiratory cycle), and in severe circumstances, (2) relatively non-varying increases in chest impedance can detect venous air emboli using non-invasive or minimally invasive continuous techniques. This would allow for automated reporting of these findings, which would be of use to anesthesia providers. The current tools (primarily echocardiogram and precordial Doppler) lack the ability to provide automated or centralized alarm functions and can be technically difficult, more costly to perform, and provide non-continuous information. A proof-of-concept open-source software tool has been developed to allow for collaborative development of this technique, and preliminary testing has been performed on a previously described cadaveric model for venous air embolism, with a single cadaver as a pilot study.

## Introduction

Venous air embolism (VAE) remains one of the leading intraoperative neurosurgical complications and causes of intraoperative neurosurgical death [1]. While transesophageal echocardiogram [2,3] and precordial Doppler are commonly used in neurosurgical cases to detect entrained air, both have significant technical limitations, and neither tool has been significantly adapted to automated notification. No currently available method provides automated alarm capability or central monitoring for VAE, and the effort and cost to use these existing methods in an anesthesia case is not insignificant. Also, many cases of significant morbidity and mortality from VAE have occurred due to hemodynamic compromise that occurred prior to any detection of its occurrence. Our group has previously developed a cadaveric model [4] to allow for rapid preclinical development of VAE monitoring tools and techniques without the need for live animal testing, given cost and increasing ethical concerns.

Chest impedance has been historically used to detect the respiratory cycle [5-7], predict minute ventilation [5], and in other contemporary applications, including heart failure management (where, for example, it has been noted that each liter of increased systemic volume decreases chest impedance approximately 2 W) [8-10]. Chest impedance uses a high-frequency 20 kHz to 100 kHz AC current to determine the chest impedance as an average single numerical value, historically at end expiration or qualitatively throughout the respiratory cycle. While the lungs only transmit approximately 5% of the current in chest impedance measurements, variations in air volume in the lungs are essentially all of the time-varying signal in normal controls. For example, when test subjects had their mouths occluded, even with attempts to breathe, no varying signal was detected despite considerable muscular activation [5,10]. (Of note are the excellent reviews on this historical topic by Van De Water et al. [11] and Baker [10].) We theorize that the remaining signal, with modern computational analyses, would be useful for minimally invasive automated detection of VAE - though this remains to be proven.

Contemporary investigators have also developed tomographic methods [12] to map out tissue density in three dimensions using arrays of impedance sensors, which have been used in research environments to study acute respiratory distress syndrome [13,14] and exercise physiology as well. With the exception of automated respiratory rate monitoring, chest impedance monitoring has yet to become a routine clinical tool; however, despite its portability, low cost, and safety, as imaging resolution is greatly dwarfed by other radiographic methods [15,16]. Many of the advanced mathematical techniques [17] used in computed (radiation) tomography are also applicable to electrical impedance tomography, though they do not appear to be necessary or useful for the detection of VAE.

The frequency used to determine the impedance determines the maximal safe current allowed to avoid any risk of cardiac arrhythmia [10] or impairment in spontaneous respiratory function. Medical standards dictate the maximum allowable current that may be safely injected into a patient, starting at 50 μA root mean square (RMS) at direct current (DC, 0 Hz) through 1 kHz. The allowable current then doubles for every doubling in frequency, increasing to 1 mA at 100 kHz, and then remains constant at 1 mA. (At the frequencies of 50 to 100 kHz used in these studies, however, approximately 30 mA would be required to stimulate a ganglion cell.)

## Technical report

A fresh frozen cadaver, including head, neck, chest, abdomen, and pelvis, was obtained via our hospital’s anatomical bequest program (Department of Anatomy, Mayo Clinic, Rochester, Minnesota, USA), which had previously been exsanguinated but not chemically preserved. The individual had bequeathed his body for the purposes of medical research and education at our institution. After our experimentation, which took place from February 22 to 24, 2023, the specimen was used for educational dissection by medical students and other surgical teams at our institution and was ultimately cremated and honored in an annual ceremony performed by our anatomical lab with the families of the recent donors. Additionally, the experimentation was approved by the Mayo Clinic Institutional Review Board Biospecimens Subcommittee under study ID 22-008554, which did not require explicit written consent given the nature of the anatomical bequest.

A proof-of-concept open-source software tool was developed in Python (Python Software Foundation, Fredericksburg, VA, US) for near real-time automated analysis of chest impedance monitoring signals (see Appendix A). Using a fresh-prepared human cadaveric torso and a reversed cardiac bypass dissection protocol, which allowed an approximation to selective cardiac and pulmonary perfusion using normal saline [4], we instilled 60 mL aliquots of air into the inferior vena cava (IVC) during simulated perfusion with and without mechanical ventilation and created digital recordings of the air entrainment.

**Figure 1:**
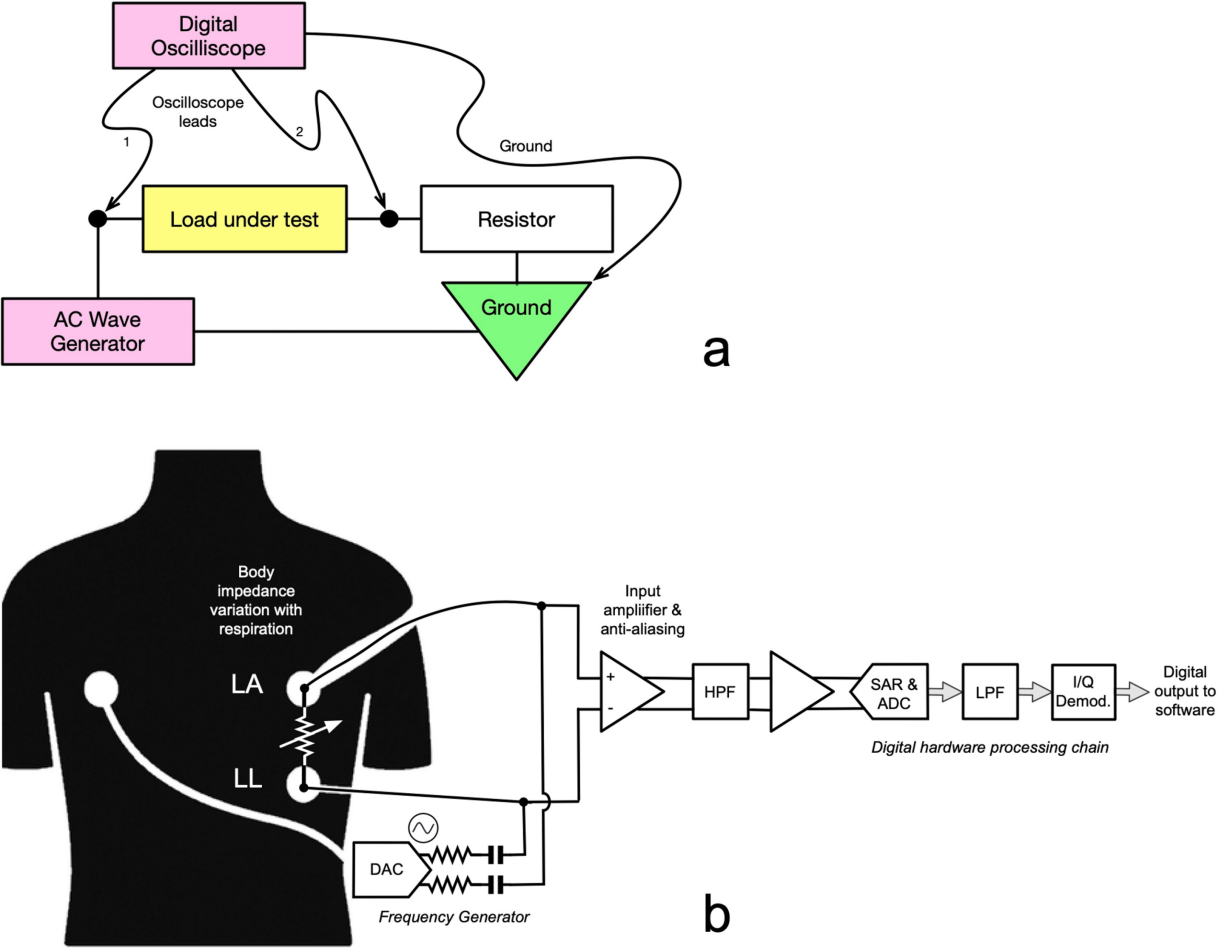
Impedance measurement apparatus (a) Simplified investigational analysis circuit used with a computerized oscilloscope. Using Ohm’s law, assuming a constant voltage AC current provided by the oscilloscope’s waveform generator, the impedance (Z) of the load can be measured using the two scope voltages and the capacitance-free physical resistor value (R), with the formula Z = V_load_ * R/V_resistor_. The value V_load_ is the difference in the two reference voltages of the oscilloscope leads 1 and 2, and V_resistor_ is the difference between leads 2 and ground. A typical R value for this application would be 100 W, and generator voltages between 0.5 and 3 volts would be used, depending on total circuit impedance to limit currents to non-hazardous levels. (b) Typical signal chain for modern high-resolution impedance pneumography measurement, with independent drive and measure paths as would be designed into a commercial integrated circuit (such as those found in an ICU monitor) [18]. LA: left arm; LL: left leg; DAC: digital-to-analog converter; HPF: high pass filter; SAR: successive approximation register; ADC: analog-to-digital converter; LPF: low-pass filter; I/Q Demod.: in-phase and quadrature signal demodulator Credit: Figure 1a was created by the authors.

The monitoring apparatus consisted of two 26-gauge neuromonitoring needles soldered to 22 American Wire Gauge (AWG) unshielded copper wires, which were connected to a laptop computer-powered digital oscilloscope, the Analog Discovery 2 digital oscilloscope (Diligent Corp., Pullman, Washington, USA), using the impedance attachment board (see schematic diagram in Figure 1a). This is conceptually similar to commercially available high-resolution integrated circuits designed for respiratory rate monitoring and other similar applications (Figure 1b). Using a fixed load resistor, the AC voltage drop was measured across the sample needle leads at a stimulation frequency of 50 kHz, which was sampled 32 times and averaged every 10 ms, and recorded to a computer file. The needle pairs were sequentially inserted into the lateral aspects of the chest wall into the subcutaneous space, and later, the esophagus, diaphragm, and pulmonary artery, where recordings were performed. While this approach could be extended with a sufficient signal-to-noise (SNR) ratio to external leads adhesive to skin, invasive needles eliminate the resistance of skin to increase the SNR, given the prototype hardware limitations.

The design of the software tool was to detect two major signals of air entrainment: (1) rapid, sudden, brief increases in impedance or “blips” representing large air boluses, which are generally transient and higher risk for false positives, with large changes lasting a few seconds or less; or (2) more gradual increases in impedance consistent with slow entrainment of air in the setting of otherwise stable respiratory variation. The tool evaluates for matched increases in both end-inspiratory and end-expiratory impedance.

**Figure 2:**
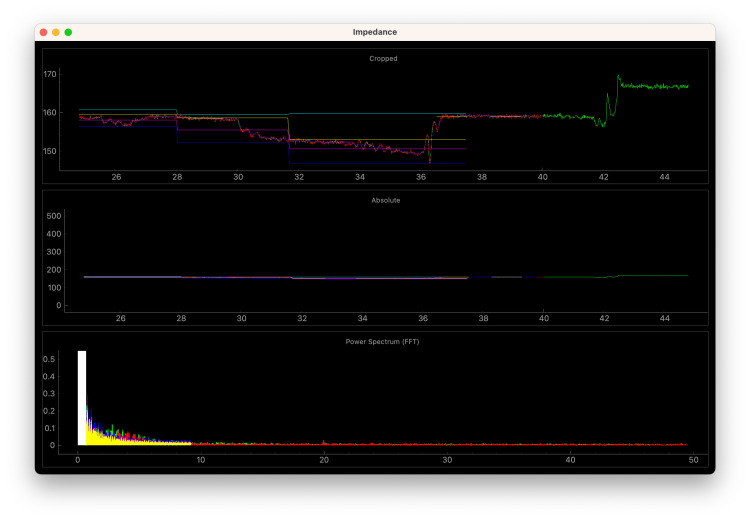
Graphic user interface screen capture from cadaveric recording of respiratory cycle during mechanical ventilation A screen capture of a period of mechanical ventilation of a cadaveric model (data originally captured on February 24, 2023) in a cadaveric model under “reversed” cardiac bypass [4]. The green waveform depicts the original captured data, which is then smoothed and shown as the red waveform. This is then isolated into separate respiratory peaks with maximum, 25th percentile, 75th percentile, and minimum values in that respiratory cycle depicted by the teal, yellow, magenta, and blue lines.

This scheme inherently relies on respiratory cycle detection. While many simple algorithms can be conceived to track the underlying respiratory cycle in the impedance waveform data, the software tool performs resampling to obtain a time-delayed running average and then performs wavelet peak detection technique [19] to identify the peaks of the respiratory cycle (which corresponds to end-inspiration), which are compared to an expected interval for surgical mechanical (or spontaneous) ventilation of 6 to 30 breaths per minute. This yielded fewer false positives than more basic minima/maxima techniques. The waveforms are then separated into individual respiratory cycles, and mathematical comparisons are performed on the 25% and 75% percentile values to attempt to determine the relative changes in impedance independent of respiratory variation (Figure 2). When there are increases in both end-inspiratory and end-expiratory impedance, air entrainment is assumed (as opposed to isolated changes in either, which would more likely represent positioning or mechanical ventilation-related changes).

**Figure 3:**
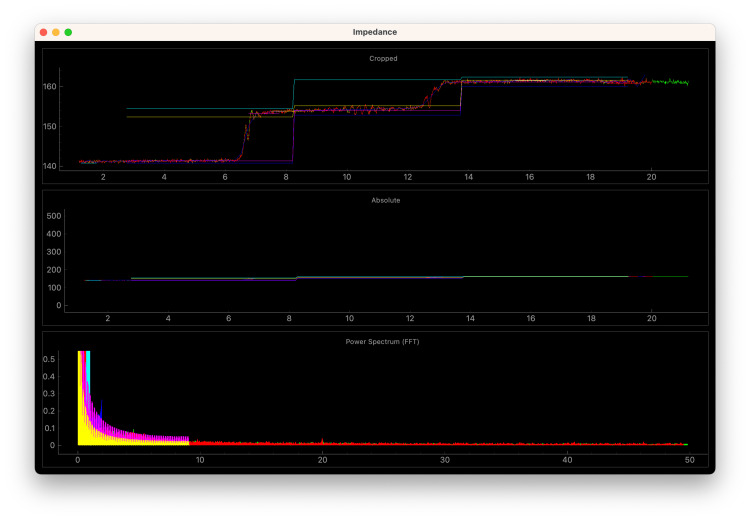
Graphic user interface screen capture from cadaveric recording of air embolism without mechanical ventilation A screen capture of a period including two injections of 60 mL of air via central venous access during “reversed” cardiac bypass of a cadaveric model (data originally captured on February 24, 2023). The software is incorrectly isolating respiratory cycles in the absence of mechanical ventilation, which was expected for this test case, given our inability to ventilate and circulate our cadaver simultaneously [4]. However, the absolute increase in impedance causes the VAE score to exceed the threshold and an audible alarm to occur accordingly (as depicted in the video in Appendix A). VAE: venous air embolism

**Figure 4:**
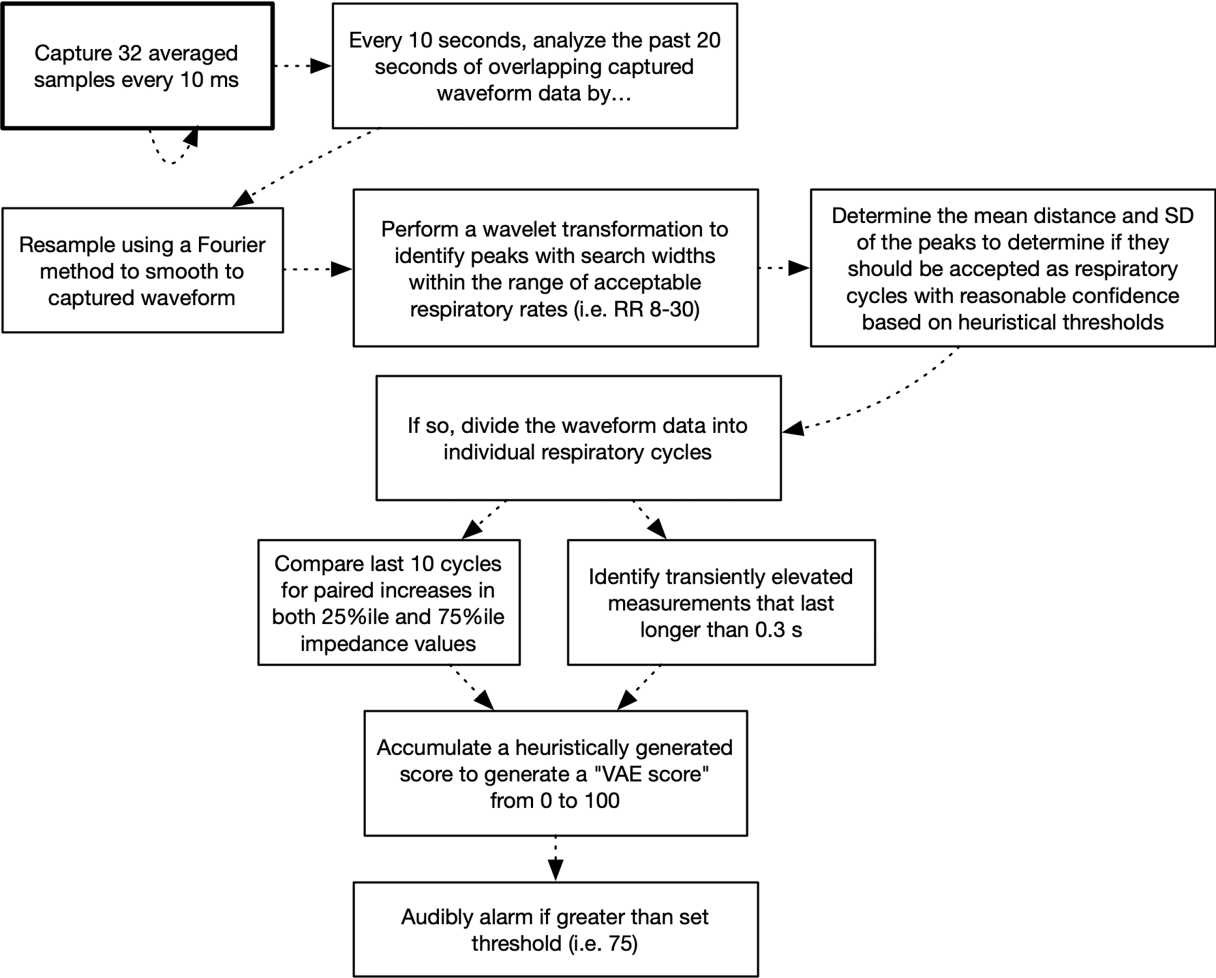
Simplified state machine for analysis of captured chest impedance data Using overlapping portions of the signal, we perform a peak-based analysis to determine the dominant respiratory frequency (as the respiratory cycle is the dominant signal in the chest impedance measurement). The presence, regularity, and amplitude of this signal are used as one of the main contributors to the signal quality index (SQI). If we find a reasonable respiratory waveform component, we then divide the period into individual periods. Then, the simplest and most accurate method for determining the end-inspiratory and end-expiratory impedance (EII, EEI) is to find the minimum and maximum of the waveform within each period. However, to reduce noise artifacts, we find the 25th percentile and 75th percentile values. (The comparison of this to the actual minimum and maximum values is also used in the calculation of the SQI.) We then take each period and compare the EEI and EII from that in the prior interval and use this to determine the likelihood of gross air entrainment. An increase in both EEI and EII as opposed to a change in either parameter alone is more suggestive of VAE (or a change in positive end-expiratory pressure, PEEP) as opposed to changes in other mechanical ventilation parameters (namely, tidal volume, or TV), which would be expected to modify only EII, or an uncommon (except perhaps in a pressure control mode) increase in PEEP combined with a decrease in TV could cause an isolated increase in EEI. Hence, paired simultaneous changes in EEI and EII will add to the VAE probability. Consistency in any of these values otherwise will add to the SQI. Irregular hand ventilation may impair this technique and will result in a poor SQI, which will indicate a lack of useful data.

Both these relative changes and sudden, brief increases are summarized into alarm signals and a score representing the probability and severity of air entrainment (Figures 3-4). To aid in clinical application, an assessment of data quality is important to help determine if the monitors are correctly connected and assess the degree of interference from surgical activity and changes in mechanical ventilation. This is summarized as a signal quality index (SQI), as is done in other automated pulse oximetry and EEG monitoring systems. This is a heuristic estimate based on a number of inputs; however, respiratory waveform detection and signal consistency are currently the major contributors to this value. The software is designed to record and replay impedance data easily for development purposes, and the raw waveform data recorder from cadaveric testing is included in the software repository (see Appendix A).

## Discussion

Multiple cadaveric simulation trials were performed (see Figure 2 and Appendix A). Differences in needle placement affected results and are the main factor in the usefulness of this technique, though ultrasound may be a useful adjunct to invasive needle placement. More invasive monitoring locations, such as between the right atrium (RA) and esophagus, yielded the most convincing waveforms and alarm data. We suspect that this would be analogous to recording with monitoring leads introduced via or placed on a central line catheter and part of an orogastric tube, respectively.

Despite the little intrinsic time variation in chest impedance beyond the respiratory cycle and, we theorize, VAE, in order to allow for adequate detection of minute variations in impedance, the SNR must be kept as high as possible, given the significant noise that would be introduced by movement in the surgical field. Unlike our large test bolus of air (60 mL), clinically significant air embolism is expected to lead to an increase in impedance on the order of 0.01 to 1 W (as compared to the respiratory cycle which can vary from approximately 5 to 10 W depending on volumes and volume status), the total impedance of the cabling, leads, and skin needs to be kept as small as possible (though the exact requirement for optimal resolution is unknown). The greatest contribution to this is the skin, which has a strong frequency dependence, which becomes satisfactorily low near 100 kHz (approximately 220 W for unprepared clean skin [20], though still yielding an SNR of 1:1000, which will pose a significant but not unsurmountable challenge for reliable measurement given the resolution of modern digital-to-analog converters (DACs) and circuit designs) (see Figure 1) [18]. Higher frequencies (above 100 kHz) are not likely to be practical as stray capacitances can be difficult to control, and interference from sources such as surgical equipment will become problematic [18]. Skin preparation, including degreasing and mild abrasion, can improve this impedance, and many technological advancements previously applied to electroencephalography (EEG) leads may be helpful. Pre-gelled abrasive silver-silver chloride leads, such as the Medtronic Zipprep^TM^ system (Minneapolis, MN, USA) for the BIS Quattro sensor, may be worthwhile to test. A pair of small monitoring needles (e.g., 25-gauge short-length solid-core steel needles connected to wire leads), while not strictly non-invasive, will provide better SNR by eliminating this impedance and may be necessary to achieve our intended results, and will be the first apparatus studied. More invasive techniques, such as RA to esophageal monitoring, would be less affected by respiratory variation given their central location and electrical isolation of the vasculature.

There are many limitations in this preliminary analysis. The volumes used to permit detection (60 mL) are much higher than an ideal system would require; however, they are still in the realm of clinically useful air volume detection. It is unclear if this technique could ever be adapted to smaller volumes. Also, the two arbitrary modalities of detection were chosen arbitrarily and may not represent an ideal scheme for the detection of air with impedance. Our analysis technique may also be highly susceptible to artifacts (e.g., irregular hand ventilation, arrhythmias, high electrocautery noise). The use of a single patient and limitations in controlled conditions preclude statistical analysis, though our main point in this publication is to share a theoretical methodology as opposed to demonstrating conclusive effectiveness. Medical device validation from an effectiveness perspective (as opposed to safety) may be difficult to establish. Overall, there is considerable work required to determine if this is a useful and viable technique, though we hope this preliminary discussion spurs further research into this topic.

## Conclusions

We speculate that impedance may be a useful tool to automate monitoring of VAE and help in its detection in ambiguous situations, or in cases where conventional monitoring is not available. Detection of false negatives from suboptimal monitor positioning is a difficult problem, and awareness of clinical status will remain important for any electrical technique, though larger arrays of electrodes or other modalities of detection may ameliorate this. Robust respiratory detection is also a formidable problem in the face of surgical activity and patient movement, and an area of future investigation. Certainly, there is considerable room for improvement in the automated detection of air embolism using chest impedance, and a need to explore multidetector techniques as well. Developing an artificial neural network classifier for respiratory detection and noise suppression based on a large body of training data may prove the optimal model, if this can be captured from high-fidelity data, whether it be from a cadaveric model or non-invasive detection during surgery under a research protocol. We are hopeful that our preliminary work will lead to future advances in VAE detection and improved patient safety in high-risk surgeries.

## Appendices

Appendix A
